# The protective effect of allium cepa against ethylene glycol-induced kidney stones in rats

**DOI:** 10.1016/j.heliyon.2023.e21221

**Published:** 2023-10-20

**Authors:** Reham M. Wahid, Nancy Husseiny Hassan, Walaa Samy, Eman Mahmoud Faragallah, Nanees F. El-Malkey, Aliaa Talaat, Alia Ghoneum, Dara Aldisi, Mahmoud M. Malek

**Affiliations:** a Physiology, Faculty of Medicine, Zagazig University, Egypt; b Human Anatomy and Embryology, Faculty of Medicine, Zagazig University, Egypt; c Medical Biochemistry, Faculty of Medicine, Zagazig University, Egypt; d School of Medicine, Wake Forest University, Winston Salem, NC, USA; e Community Health Sciences Department, College of Applied Medical Sciences, King Saud University, Riyadh 11433, Saudi Arabia; f Urology and Andrology, Faculty of Medicine, Zagazig University, Egypt

**Keywords:** Kidney stones, allium cepa, Oxidative stress, Inflammatory markers, Autophagy, Osteopontin

## Abstract

**1)Background:**

Kidney stones is one of the serious medical conditions affecting populations worldwide. So, we aimed in this study to investigate the protective effect of allium cepa administration against KSD.

**2)Methods:**

24 adult male albino rats were assigned into 3 groups; group I: control group; group II: received ethylene glycol (EG) in the drinking water for 4 weeks; and group III received EG in the drinking water plus freshly prepared allium cepa extract (ACE) for 4 weeks. Renal function tests and urine analysis were done. Tissue oxidative stress markers (SOD and MDA) were assessed, and kidney expression of SIRT-1, Beclin, LC3, osteopontin, and Regucalcin were measured by RT-qPCR. Histopathological assessment and immunohistochemistry for Bax, Beclin-1 and TNF-α were performed.

**3)Results:**

There was a significant improved kidney function tests in the ACE received group compared to EG group (P < 0.001). The present study showed less stones formation and apoptosis with decreased osteopontin and autophagy genes expression in the ACE received group compared to EG group (P < 0.001). While, regucalcin and SIRT-1 genes showed higher expression in the former group than the later group (P < 0.001).

**4) Conclusion:**

Alium Cepa extract administration has a significant protective effect against kidney stones formation.

## 1 Introduction

Kidney stones are recognized as a significant medical issue with a high prevalence, impacting approximately 12 % of the global population [1]. The most prevalent form of stone formation is calcium stones, constituting 80 % of cases; calcium oxalate is notably the primary component of this type [2]. In spite of the availability of recent technologies and advanced management methods for kidney stones (such as percutaneous nephrolithotomy and lithotripsy), numerous unfavorable consequences and undesired effects arise from these procedures. These include kidney injuries, an increased likelihood of stone recurrence, and diminished renal function post-procedure. Additionally, the considerable cost and ineffective outcomes associated with other treatment modalities further compound these issues [3]. Consequently, there is a pressing need to explore alternative approaches for both the prophylaxis and treatment of kidney stones.

Numerous research studies have demonstrated that exposure to high levels of oxalates results in an escalation of reactive oxygen species (ROS) and subsequent peroxidative damage [4]. As a consequence, oxidative stress is widely recognized as a fundamental underlying mechanism in the process of stone formation. This mechanism encompasses various stages, including the initiation of crystals, their growth, aggregation, and eventual retention within the renal tubules [5]. An accumulating body of evidence suggests that the aforementioned crystallization process can be counteracted by the presence of stone inhibitors within urine. Nevertheless, there exists a significant potential for stone formation when the delicate equilibrium between crystallization and the presence of stone inhibitors is disrupted [6].

Furthermore, a heightened concentration of calcium oxalate crystals has a toxic effect on renal epithelial cells, leading to their injury and triggering apoptosis. This, in turn, renders the cellular surfaces more susceptible, thereby enhancing the adhesion and attachment of crystals. This chain of events further exacerbates epithelial damage, particularly in the presence of crucial factors like oxidative stress [2]. Recent literature has illuminated the role of antioxidants and anti-inflammatory agents as viable alternatives for safeguarding against the development of kidney stones. This highlights the dire need for natural compounds that can offer a secure, efficacious, and economically feasible approach to managing the formation of kidney stones.

Onion (allium cepa), a widely consumed vegetable belonging to the Liliaceae family, holds global popularity and serves as a common culinary ingredient. Its diverse array of roles—ranging from antifungal, anticancer, anti-inflammatory, antioxidant, antimicrobial, and antimutagenic to antidiabetic and antiplatelet activities—has been extensively examined through various research studies [7]. These multifarious benefits can be attributed to its key constituents, including flavonoids, glycosides, anthocyanins, allicin, and quercetin [8]. Hence, the objective of our study was to explore the protective effect of allium cepa administration against the process of kidney stone formation and its associated impact on tissue injury.

## 2 Methods

### 2.1 Experimental animals and design

This study was conducted on 24 adult healthy male of local strain albino rats weighing 190–240 gm. The rats had free access to water and chow, and were kept at room temperature and stayed on a 12 h light/dark cycle. The experimental protocol was approved by Zagazig university institutional animal care and use committee (ZU-IACUC) with number ZU-IACUC/2/F/65/2022, and in accordance with the National Institutes of Health guide for the care and use of Laboratory animals (NIH Publications No. 8023, revised 1978)

Rats were divided into 3 groups with 8 rats in each; Group I (control group) in which rats got normal diet and drinking water, and were administered 5 ml/kg distilled water orally, group II (kidney stones group) in which rats received 0.75 % ethylene glycol in the drinking water, and were administered 5 ml/kg distilled water orally for 4 weeks [9], and group III in which rats received 0.75 % ethylene glycol in the drinking water plus freshly prepared allium cepa extract (ACE) 5 ml/kg administration using intra-gastric tube for 4 weeks [10]. ACE was prepared according to the method described by Refs. [10,11]**.** At the end of the 4th week, each rat was housed a metabolic cage to collect 24-h urine with assessment of urine volume, rats then were anaesthetized using pentobarbital sodium 50 mg/kg, intraperitoneal, blood samples were collected by orbital veins, at the end of the study, rats were sacrificed and both kidneys were removed to use them in histopathology and RT-PCR.

### 2.2 Urine analysis

Creatinine level was measured using commercial assay kits (Spinreact, Spain), then kidney injury molecule-1 (KIM-1) were analyzed by KIM-1 ELISA kit (Jianglai, China). The concentrations of total protein in urine was determined using assay kits (Bioassay systems, USA) and the total protein output was calculated by protein conc. multiplied by urine volume/24 h.

### 2.3 Biochemical analysis

The concentrations of serum creatinine and blood urea nitrogen (BUN) were detected using commercial assay kits (Spinreact, Spain), then creatinine clearance was calculated using the following equation; creatinine clearance = urine volume * urine creatinine/plasma creatinine.

### 2.4 Assessment of antioxidant activity and lipid perioxidation level

SOD activity and MDA level were measured spectrophotometrically in the kidney homogenate using (Biodiagnostic kit) according to the instructions of the manufacturer [12], [13].

### 2.5 Gene expression analyses

We homogenized renal tissue samples and extracted total RNA using the RNeasy Mini Kit from Qiagen, following the manufacturer's protocol. We checked the purity of the RNA by measuring the 260/280 nm absorption ratio, which was between 1.8 and 2.0 for all samples. We then used the QuantiTect Reverse Transcription Kit to create cDNA from the RNA, and analyzed gene expression using qRT-PCR with a 5 μL of the cDNA, 10 pmol/uL of each primer, 10 μL of SYBR™ Green PCR Master Mix (Applied Biosystems™). RT-qPCR was performed by Mx3005P (Stratagene, CA, USA). We performed thermal cycling with a denaturation step at 94 °C for 5 min, followed by 40 cycles of 94 °C for 30 s, annealing at specific temperatures for 30 s, and elongation at 72 °C for 30 s, with a final extension step at 72 °C for 10 min. We normalized the data to GADPH transcript levels and used the 2-ΔΔCt method to calculate the relative expression of the genes [14]. The sequences of the primers used in RT-qPCR are listed in Table (1).

**Table (1):** primer sequences of Gapdh, Beclin-1, LC-3, SIRT-1, Osteopontin and Regucalcin.

Gene	Forward primer	reverse primer	tm	bp	Accession no.
Gapdh	5′GCATCTTCTTGTGCAGTGCC3′	5′TACGGCCAAATCCGTTCACA3′	55	74	NM_017008.4
Beclin-1	5′GAATGGAGGGGTCTAAGGCG3′	5′CTTCCTCCTGGCTCTCTCT3′	55	180	NM_001034117.1
LC-3	5′GAGAGCGAGAGAGATGAAG 3′	5′CGGATAGTCTAGTTTAGATGAG 3′	53	266	NM_022867.2
SIRT-1	5′TGTTTCCTGTGGGATACCTGA3′	5′TGAAGAATGGTCTTGGGTCTT 3′	58	137	NM_001372090.1
Osteopontin	5′ATGGCTTTCATTGGAGT TGC3′	5′GAGGAGAAGGCGCATTACA G3′	51	165	NM_012881.2
Regucalcin	5′GGAGGCTATGTTGCCACCATTGGA3′	5′CCCTCCAAAGCAGCATGAAGTTG3′	62	558	NM_031546.2

### 2.6 Histological assessment by light microscopic examination

#### 2.6.1 Crystal counting

Six fields of each section, made up of three fields in the medulla and three fields in the cortex, were examined using a light microscope with a magnification of 400x to examine the histopathological alterations and calcium oxalate crystal formation. In 54 microscopic fields, crystal deposits accumulated in the renal tubules were counted, and an average was determined using ImageJ (Image analyzer) (Fiji image j; 1.51 n, NIH, USA), the size of the crystal deposition was determined and classified as zero (no crystal), small (1–10 μg), medium (10–20 μg), and giant (>20 μg)).

Additionally, each kidney was assessed for crystal deposition using the following scoring criteria in order to undertake statistical analysis: No crystals are represented by 0, small crystals by 1, medium crystals by 2, and huge crystals by 3 [15].

### 2.7 Histopathological characters by hematoxylin and eosin stain

The samples from each group were processed at the Pathology Department of the Faculty of Medicine at Zagazig University. They were fixed in Bouin's solution, turned into paraffin blocks, and cut into sections 5 micrometers thick. The sections were then mounted on glass slides, de-paraffinized using xylene, and stained using both hematoxylin and eosin (H&E).

### 2.8 Histopathological characters by Toluidine Blue stain

The renal cortex samples (1 mm thickness) were post-fixed in 1 % osmium tetra-oxide, dried, and fixed in resin after being preserved in a mixture of 2.5 % glutaraldehyde and 2.5 % paraformaldehyde for 24 h. The samples were divided into portions that were semi-thin. Toluidine blue was used to stain semi-thin Sections (1 μm), which were then examined under a light microscope.

Immunohistochemistry (IHC) for Beclin-1 (BECN-1), (BCL2-Associated X Protein) BAX & Tumor Necrosis Factor α (TNF-α)

We used Bouin-fixed renal samples that had been turned into paraffin blocks for immune-histochemical analysis. The paraffin sections were 4 mm thick, mounted on positively charged slides, deparaffinized in xylene, hydrated using decreasing concentrations of ethanol, and treated with 3 % hydrogen peroxide in methanol to inhibit endogenous peroxidase activity.

The samples were treated with 3 % H2O2 for 15 min at 37C to block endogenous peroxidase activity, and broad binding was blocked with 5 % bovine serum albumin at room temperature for 20 min. Finally, an overnight specimen incubation with antibodies against the beclin-1 (1:100), anti-Bax, rat monoclonal antibody (1:50, no. 13401A, clone G206-1276, immunoglobulin [Ig] M, 0.5 mg/mL, PharMingen, San Diego, CA) and tumor necrosis factor -alpha (TNF - α) (catalog No. EK0526; BosterBio, Pleasanton, USA) was done.

Then, the renal samples were washed using PBS then a direct incubation with the secondary antibody (1:500 in PBS) for 30 min. By the end, antibody binding was visualized using a diaminobenzidine kit.

### 2.9 Histopathological scoring

To evaluate the severity of the damage, renal sections were examined and scored. The endothelial, glomerular, tubular, and interstitial (EGTI) scoring method, which was specifically designed for animal investigations on kidney tissue in the setting of injury, was used to evaluate histological damage and measure it by a qualified histo-pathologist who was blinded to the group allocation. In all groups of the renal cortex that were evaluated, the scoring method was done [16].

### 2.10 Histo-morphometric analysis

Morphometric analysis was performed on each rat in each group. Mast cells were detected using Toluidine blue staining as previously described. Mast cells were identified using purple granules, and activated mast cells were characterized by granules that had been discharged and loosely packed. Activated mast cells per field were counted at a magnification of 400x. After staining for BECN-1, the density of immune-reactive structures was evaluated based on optical density (OD), which was calculated using the formula: OD = log (256/mean gray level). The background was subtracted and the OD of the image file was calibrated as a percentage (relative optical density, ROD) using Image J analysis software at the Human Anatomy and Embryology Department at Zagazig University. In addition, in sections stained for BAX and TNF-α, representative fields across the pictures captured by the light microscope at 400x magnification were chosen to quantify the percentage of positive response in the renal cortex and medulla from 8 rats per group. To quantitatively analyze the immunoreactivity, the stained sections were examined and analyzed by light microscopy (LEICA ICC50 W) in the Image Analysis Unit of the Human Anatomy and Embryology Department at the Faculty of Medicine at Zagazig University.

### 2.11 Statistical analysis

Continuous variables were expressed in mean ± SD. Normality was patterned by the Kolmogorov-Smirnov test. The statistical analysis was done using SPSS program (19) (SPSS Inc. Chicago, IL, USA) and one-way ANOVA was used to assess changes among groups where p value ≤ 0.05 was considered significant. The median and interquartile range (IQR) were used to state non continuous data. Moreover in that case, the Kruskal-Wallis test and Dunn's multiple comparison test were applied.

## 3 Results

### 3.1 Hemodynamics, glomerular and tubular function

It was noticed that there was a significant elevation of creatinine clearance in the treated group compared to EG group (P < 0.05). However, there was a significant decrease of the same parameter compared to the control group (P < 0.05) Table (2). In addition, serum creatinine and urea levels, total protein excretion and KIM level in urine were significantly decreased in the treated group compared to EG group (P < 0.001). On other hand, these parameters showed higher values compared to the control group Table (2).

**Table (2):** Renal function tests and urine analysis findings in all studied groups.

	group I	group II	group III	F	P
Serum creatinine level mg/dl	0.65 ± 0.09	2.9 ± 0.33^a^	0.86 ± 0.13^ab^	276.6	<0.001**
BUN level mg/dl	16.1 ± 3.08	67.89 ± 6.3^a^	23.5 ± 4.7^ab^	259.03	<0.001**
Creatinine clearance ml/min	0.55 ± 0.36	0.05 ± 0.02^a^	0.3 ± 0.09^ab^	10.65	0.001**
Proteinuria mg/dl	85.2 ± 12.7	247.12 ± 18.8^a^	123.5 ± 15.29^ab^	237.8	<0.001**
protein output in urine mg/24h	3.06 ± 0.46	9.5 ± 0.83^a^	4.6 ± 0.62^ab^	209.6	<0.001**
KIM level in urine ng/ml	1.1 ± 0.2	19.7 ± 2.3^a^	3.6 ± 0.75^ab^	387.4	<0.001**

All continuous variables were expressed using mean (±SD) and compared using Anova test and post hoc LSD test where.

➢ *P* value < 0.001** was considered statistically highly significant (S).

➢ ^a^ means: significant compared with group I.

➢ ^b^ means: significant compared with group II.

### 3.2 Oxidative stress markers

**Fig. 1:**
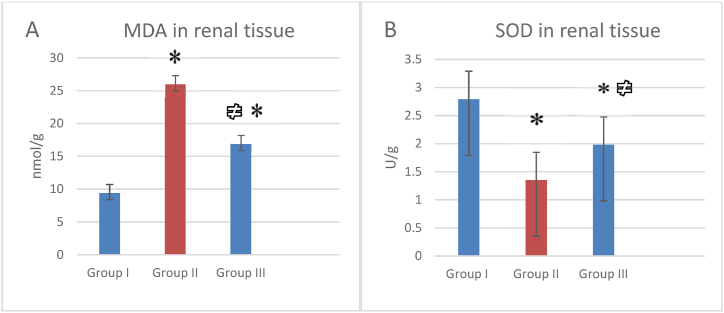
means ± SD of oxidative stress markers, (A): MDA levels in renal tissue, and (B): SOD levels in renal tissue. Statistical analysis was done using ANOVA test and post-hoc test among groups, * means P < 0.05 in comparison with the control group I, and # means P < 0.05 in comparison with the kidney stone group II.

MDA level was significantly lower in the treated group than its level in EG group (P < 0.001) figure 1a. However, SOD was elevated in the treated group compared to EG group with significant difference (P < 0.05) figure 1b.

### 3.3 Genes expression findings

The treated group showed significantly higher expression of regucalcin and lower expression of osteopontin genes than that of the EG group (P < 0.001), while it showed significantly lower expression of regucalcin and higher expression of osteopontin genes than the control group (P < 0.001). Furthermore, there was a significant decrease of autophagy genes expression (LC3 and Beclin) in the treated group compared to the EG group, with the highest levels in the later group compared to all studied groups (P < 0.001) Table (3).

It was observed that SIRT gene expression was the highest in the EG group compared to all other groups (P < 0.001), and it showed significantly lower expression in the treated group (P < 0.001) Table (3).

**Table (3):** expression levels of Beclin-1, LC-3, SIRT-1, Osteopontin and Regucalcin genes in renal tissue in all studied groups.

	Control group I	EG group II	Treated group III	F	P
Osteopontin expression	1.24 ± 0.24	2.28 ± 0.19^a^	1.43 ± 0.16^b^	59.9	<0.001**
Regucalcin Expression	1.1 ± 0.12	0.5 ± 0.09^a^	0.85 ± 0.13^ab^	49.6	<0.001**
SIRT1 Expression	1.03 ± 0.12	0.44 ± 0.1^a^	0.78 ± 0.07^ab^	64.6	<0.001**
LC3 Expression	1.14 ± 0.09	2.44 ± 0.22^a^	1.59 ± 0.18^ab^	111.4	<0.001**
Beclin Expression	1.08 ± 0.23	2.05 ± 0.2^a^	1.50 ± 0.15^ab^	46.4	<0.001**

All continuous variables were expressed using mean (±SD) and compared using Anova test and post hoc LSD test where.

➢*P* value < 0.001** was considered statistically highly significant (S).

➢^a^ means: significant compared with group I.

➢^b^ means: significant compared with group II.

### 3.4 Tissue sampling

#### 3.4.1 Gross features results

**Fig. 2:**
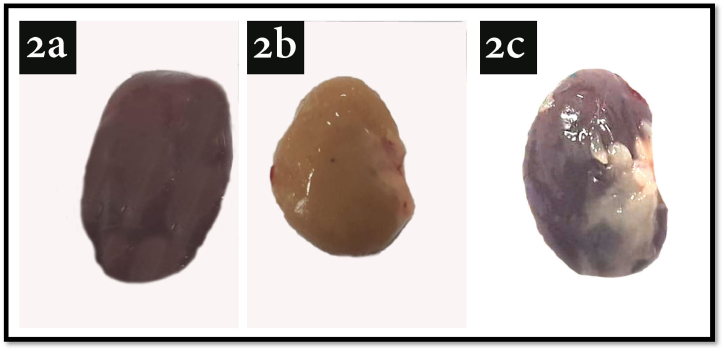
Illustrating photos show the gross features of the kidney in all groups by the end of the experiment. 2a: Group I with the normal color, average size and dimensions, 2b: Group II with marked yellowish discoloration and observed decreased all dimensions, 2c: Group III with preserved normal color of the kidney and slightly decreased dimensions. (For interpretation of the references to color in this figure legend, the reader is referred to the Web version of this article.)

The gross examination revealed marked changes in the color on the external surface of the kidneys taken from animals in groups I, II, III. (Fig. 2). Group I appeared with the normal color (Fig. 2a), average size and dimensions (Table 4). On the other hand group II was with marked yellowish discoloration (Fig. 2b) with an observed decrease in all dimensions **(**Table 4**).** Furthermore, group III revealed a preserved normal color of the kidney (Fig. 2c) and slightly decreased dimensions **(**Table 4**).**

**Table (4):** Comparison between all experimental groups’ right kidney dimensions.

*Parameters*	*Group I*	*Group II*	*Group III*	*P-value (p<0.05)*
*Length (cm)*	2.059 ± 0.17	1.024 ± 0.14 ^**a**^	1.664 ± 0.08 ^**ab**^	0.0001**
*Width (cm)*	0.877 ± 0.07	0.501 ± 0.08 ^**a**^	0.671 ± 0.05 ^**ab**^	0.0001**
*Thickness (cm)*	0.762 ± 0.05	0.437 ± 0.06 ^**a**^	0.628 ± 0.02 ^**ab**^	0.0001**

All continuous variables were expressed using mean (±SD) and compared using Anova test and post hoc LSD test where.

➢ *P* value ≤ 0.001** was considered statistically highly significant(S).

➢^a^ means: significant compared with group I.

➢^b^ means: significant compared with group II.

### 3.5 Histo-pathological assessment results

#### 3.5.1 Crystal count

Table (5), Table (6) displays the average number of crystal deposits found in the microscopic fields of kidney specimens from various groups. In group II, the mean number of deposits was larger than group III. In the control group (group I), there was no crystal deposition at all in the kidneys. To summarize the findings and offer stronger proof demonstrating that dietary supplements have a protective effect in opposition to crystal growth and emplacement, the number of identified crystals in each kidney segment or area (cortex and medulla) times the appropriate score was calculated, and the scores' mean was noted. The estimated score of crystal formation was significantly higher in group II in comparison with group III (Table (5), Table (6)).

**Table (5):** Size and the number of stone formation and crystal deposit in the cortex and medulla kidney section of the rats.

*Kidney segment*	*Cortex*			*Medulla*		
*Size*	***Small***	***Medium***	***Giant***	***Small***	***Medium***	***Giant***
*Group I*	0	0	0	0	0	0
*Group II*	20	24	10	110	57	35
*Group III*	7	2	0	10	1	1

(n = 8), p < 0.05.

Small sized crystals: (1–10 μg).

Medium sized crystals: (10–20 μg).

Giant sized crystals: (>20 μg)).

**Table (6):** Estimation of the amount of stone formation in the kidney section of the rats in different groups calculated by scoring system.

*Kidney segment*	*Cortex*	*Medulla*	*Kidney*
*Group I*	0	0	0
*Group II*	98	329	427
*Group III*	11	15	26

(n = 8), p < 0.05.

#### 3.5.2 Hematoxylin and eosin staining results

**Fig. 3:**
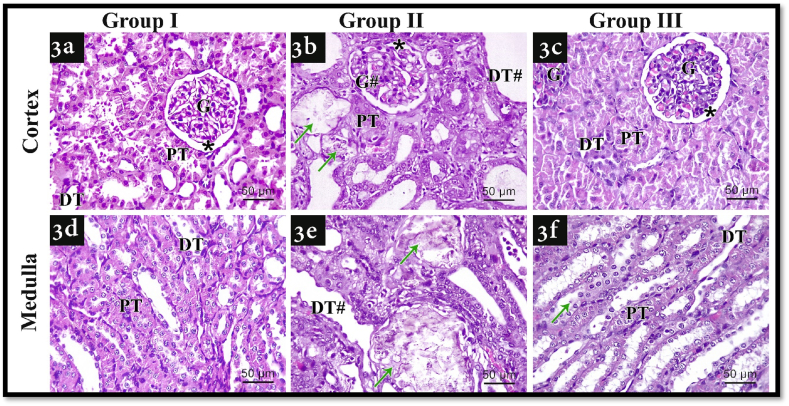
Illustrating H&E staining photomicrographs of all experimental groups. 3a) shows a cut section of renal cortex in group I, 3d) shows a cut section of renal medulla in group I, 3b) shows a cut section of renal cortex in group II, 3e) shows a cut section of renal medulla in group II, 3c) shows a cut section of renal cortex in group III, 3f) shows a cut section of renal medulla in group III. G: normal glomerulus, PT: proximal convoluted tubules, DT: distal convoluted tubules, Asterisk: Bowman's space, G#: distorted glomerulus, DT#: damaged extremely dilated distal convoluted tubules, Green arrows: kidney stones (H&E; x400, scale bar 50 μm). (For interpretation of the references to color in this figure legend, the reader is referred to the Web version of this article.)

Hematoxylin and eosin examined sections revealed distorted glomeruli with damaged extremely dilated distal convoluted tubules (DCT) in both renal cortex and medulla with apparent Kidney stones (Fig. 3b, e) in group II, compared with group I which revealed the normal microstructure of both renal cortex and medulla (Fig. 3a, d). All these damage signs were improved in an observed manner in group III (Fig. 3c, f).

#### 3.5.3 Semi-thin Toluidine Blue staining

**Fig. 4:**
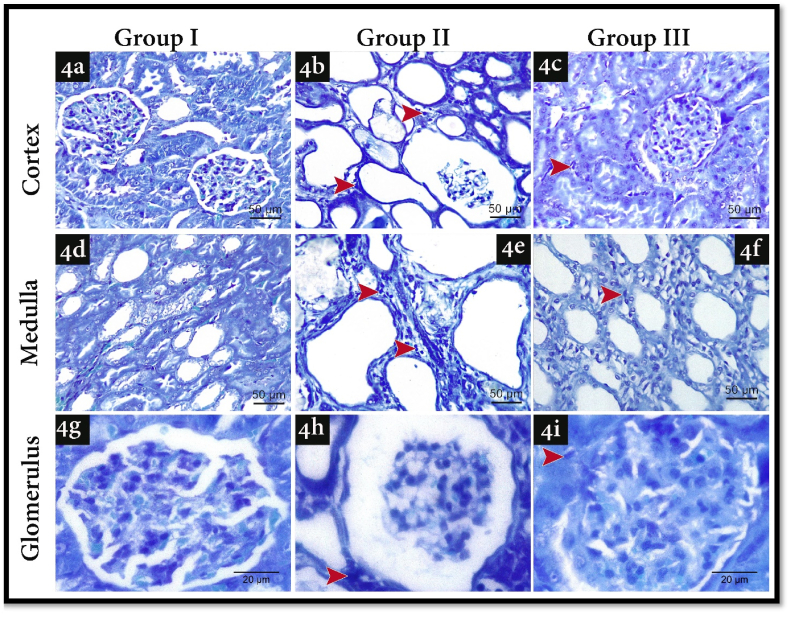
Illustrating Toluidine Blue staining photomicrographs of all experimental groups. 4a) shows a semi-thin section of renal cortex in group I, 4d) shows a semi-thin section of renal medulla in group I, 4g) shows a semi-thin section of renal glomerulus in group I, 4b) shows a semi-thin section of renal cortex in group II, 4e) shows a semi-thin section of renal medulla in group II, 4h) shows a semi-thin section of renal glomerulus in group II, 4c) shows a semi-thin section of renal cortex in group III, 4f) shows a semi-thin section of renal medulla in group III, 4i) shows a semi-thin section of renal glomerulus in group III. Red arrow heads: activated Mast cells (Toluidine Blue; a, b, c, d, e, fx400, scale bar 50 μm; g, h, I x1000, scale bar 20 μm). (For interpretation of the references to color in this figure legend, the reader is referred to the Web version of this article.)

Toluidine blue stained semi-thin sections showed that activated mast cell infiltration increased in both cortices and medullas of rats with stone formation (Group II) (Fig. 4b,e, h) in comparison to (Group I) (Fig. 4 a, d, g). Moreover, this was confirmed statistically with the significant (P ≤ 0.05) increase in mast cell number in comparison with group I (Table 8). Furthermore, in group III there was a decrease in activated mast cell infiltration (Fig. 4 c, f, i) which was confirmed by the statistically significant (P ≤ 0.05) desrease in mast cell number in comparison with group II (Table 8).

#### 3.5.4 Immune-histochemistry results

1 Beclin-1 (BENC-1) autophagy marker immune-histochemistry results

**Fig. 5:**
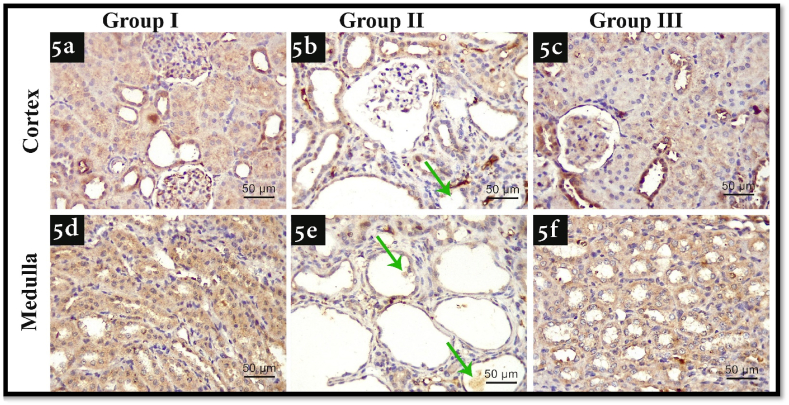
Illustrating BENC1 immunohistochemistry photomicrographs of all experimental groups. 5a, d) shows faint immune-expression of renal cortex and medulla in group I respectively, 5b, e) shows marked immune-expression of renal cortex and medulla in group II respectively, 5c, and f) shows moderate immune-expression of renal cortex and medulla in group III respectively, Green arrows: renal stones (BECN1; x400, scale bar 50 μm). (For interpretation of the references to color in this figure legend, the reader is referred to the Web version of this article.)

As shown in Fig. 5, there was an observed increase in immune-expression of BENC-1) in group II in both cortex and medulla (Fig. 5 b, e) in comparison with group 1 (Fig. 5a, d). As regards group III (Fig. 5c, f), there was an observed decrease in the expression in comparison with group II.

2 Bax immune-histochemistry results

**Fig. 6:**
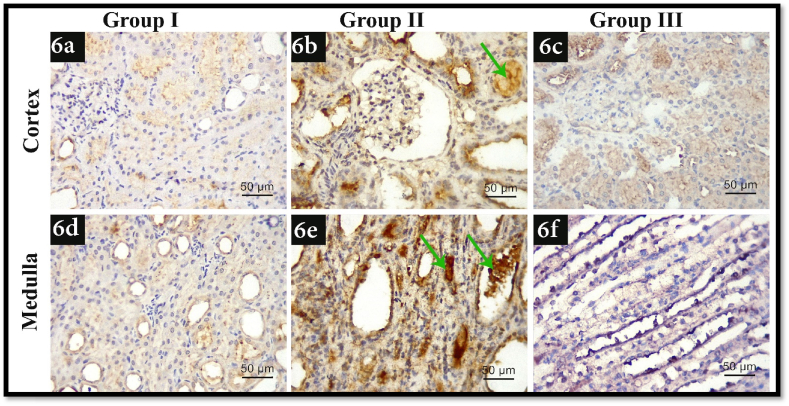
Illustrating BAX immunohistochemistry photomicrographs of all experimental groups. 6a, d) shows faint immune-expression of renal cortex and medulla in group I respectively, 6b, e) shows marked immune-expression of renal cortex and medulla in group II respectively, 6c, and f) shows moderate immune-expression of renal cortex and medulla in group III respectively, Green arrows: kidney stones (BAX; x400, scale bar 50 μm). (For interpretation of the references to color in this figure legend, the reader is referred to the Web version of this article.)

As shown in Fig. 6, there was an observed increase in immune-expression of BAX in group II in both cortex and medulla (Fig. 6 b, e) in comparison with group 1 (Fig. 6a, d). As regards group III (Fig. 6c, f), there was an observed decrease in the expression in comparison with group II.

3 TNF-α immune-histochemistry results

**Fig. 7:**
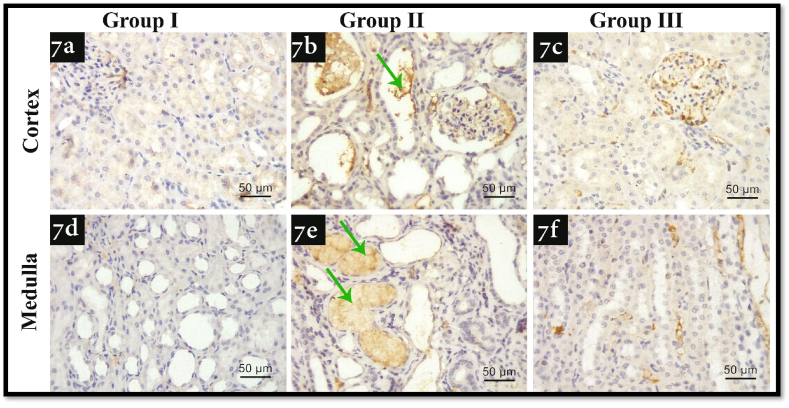
Illustrating TNF-α immunohistochemistry photomicrographs of all experimental groups. 7a, d) shows faint immune-expression of renal cortex and medulla in group I respectively, 7b, e) shows marked immune-expression of renal cortex and medulla in group II respectively, 7c, and f) shows moderate immune-expression of renal cortex and medulla in group III respectively, Green arrows: kidney stones (TNF-α; x400, scale bar 50 μm). (For interpretation of the references to color in this figure legend, the reader is referred to the Web version of this article.)

As shown in Fig. 7, there was an observed increase in immune-expression of TNF-α in group II in both cortex and medulla (Fig. 7 b, e) in comparison with group 1 (Fig. 7a, d). As regards group III (Fig. 7c, f), there was an observed decrease in the expression in comparison with group II.

#### 3.5.5 Histopathological scoring

Endothelial, glomerular, tubular, and interstitial histological damage were the four separate components that made up the scoring system. After histological analysis using light microscopy, scoring was done in all investigated groups of the renal cortex. The renal cortex of group I rats had a typical look; the brush border of the tubular cells was intact, and the basal membrane had not thickened. (Tubular score 0) No inflammation or necrosis was visible. Interstitial score of 0 indicates that there is no discernible interstitium, indicating that there is no damage or abnormality inside the interstitial compartment. The renal cortex, inflammation, and interstitial hemorrhage were all present in variable degrees in group II. In comparison to group I, it showed up in fewer than 25 % of the tissue as thicker tubular cell basal membranes, loss of the brush border in more than 25 % of tubular cells, and necrosis (Tubular score 3). The interstitial area also showed inflammation and hemorrhage, with up to 60 % of the cells showing necrosis (Interstitial score 3). The brush border of the tubular cells is unaltered in group III, and the basal membrane has only slightly thickened. Tubular score 1: Few inflammations with no necrosis can be seen. Comparatively to group II, there is no discernible interstitium, indicating that there is no damage within the interstitial compartment (Interstitial score 0, p < 0.05) (Table 7).

**Table (7):** The EGTI histo-pathological scoring results.

EGTI Damage Score	*Group I*	*Group II*	*Group III*	*P-value (p<0.05)*
Endothelial (Median (IQR))	0.0 (0.75)	4.5 (0.5)^a^	1.0 (0.0)^b^	0.0001**
Glomerular (Median (IQR))	0.0 (0.75)	7.0 (1.0) ^**a**^	4.0 (1.5) ^**b**^	0.0001**
Tubular (Median (IQR))	0.0 (0.5)	5.5 (1.25) ^**a**^	2.0 (0.75)^b^	0.0001**
Interstitial (Median (IQR))	0.0 (0.5)	7.5 (0. 5) ^**a**^	1.0 (0.75) ^**b**^	0.0001**

All variables were expressed using Median (IQR) and compared using the Kruskal-Wallis test and Dunn's multiple comparison test where.

➢*P* value ≤ 0.001** was considered statistically highly significant(S).

➢ ^a^: significant compared with group I.

➢ ^b^: significant compared with group II.

#### 3.5.6 Histo-morphometric results

As regards the mean number of activated mast cells, there was a statistically significant increase in group II in comparison with group I, while it significantly decreased in group III compared to group II. On the other hand, there was a statistically significant increase in optical density (OD) positive immune reaction of BECN-1 and area percentage of positive immune reaction of BAX and TNF-α in group II in comparison with group I, while it significantly decreased in group III compared to group II (P ≤ 0.05) **(**Table 8**).**

**Table (8):** Comparison between all experimental groups’ different morphometric parameters.

*Parameters*	*Group I*	*Group II*	*Group III*	*P-value (p<0.05)*
*Number of Activated Mast cells*	0.562 ± 0.41	7.25 ± 1.03 ^**a**^	2.75 ± 0.7 ^**ab**^	0.0001**
*BECN-1 Optical Density (OD)*	0.365 ± 0.006	0.732 ± 0.10 ^**a**^	0.419 ± 0.01 ^**b**^	0.0001**
*BAX Area Percentage (%)*	6.272 ± 0.83	27.98 ± 1.37 ^**a**^	11.71 ± 1.1 ^**ab**^	0.0001**
TNF-α Area Percentage (%)	1.809 ± 0.43	17.22 ± 0.84 ^**a**^	5.176 ± 0.66 ^**ab**^	0.0001**

All continuous variables were expressed using mean (±SD) and compared using Anova test and post hoc LSD test where.

➢*P* value ≤ 0.001** was considered statistically highly significant(S).

➢^a^ means: significant compared with group I.

➢^b^ means: significant compared with group II.

## 4 Discussion

The global prevalence of kidney stones is substantial, affecting approximately 12 % of populations worldwide [1]. Extensive literature has delved into the fundamental mechanisms underlying the formation of renal stones, revealing that it arises due to an imbalance between stone inhibitors within urine and the crystallization process [6]. This crystallization exacerbates cellular injury and death, consequently promoting greater adhesion, nucleation of crystals, and establishing a self-perpetuating cycle within the stone formation process. Furthermore, additional studies propose the presence of another intricate cycle contributing to this phenomenon. These studies underscore the pivotal role of oxidative stress as an initiating factor, inciting injury to renal epithelial cells by generating excessive reactive oxygen species, which in turn triggers a sequence of peroxidative damages, amplifying crystallization and renal injury [2]. Inflammation, a principal mechanism stemming from the impact of calcium oxalate crystals on the injury of renal epithelial cells, emerges as the most prevalent outcome of oxidative stress. Reactive oxygen species directly or indirectly fuel the inflammatory process, provoking the release of pro-inflammatory cytokines and chemokines. These molecules act as stress response triggers, thereby intensifying the cascade of inflammatory processes and contributing to renal impairment [17,18].

The present study showed kidney function impairment, evident through elevated levels of serum creatinine, BUN, and urinary KIM in the group treated with EG (experimental group). This group also exhibited heightened proteinuria and reduced creatinine clearance, indicative of impaired glomerular and renal filtration functions. Additionally, significant damage and dilation of the distal convoluted tubules, accompanied by increased deposition of calcium oxalate crystals and stone formation, were observed. In parallel, an examination of oxidative stress and inflammatory markers revealed their highest levels in the EG treated group, further emphasizing their pivotal roles in the progression of renal impairment, as highlighted in previous studies.

Numerous literatures have revealed the significant role of autophagy in kidney protection, particularly in response to specific levels of reactive oxygen species (ROS), which facilitate cellular homeostasis and repair [19]. However, the relationship between oxidative stress and autophagy is notably intricate; while moderate oxidative stress might induce autophagy as a cellular attempt at repair [19], in certain cases, like the present study, it may fail to compensate and eventually culminate in cell death. This phenomenon is underscored by elevated expression of autophagy genes Beclin and LC3 in the renal tissue of the EG group, compared with the group receiving allium cepa extract, which displayed lesser expression of autophagy markers.

Considering the high prevalence rate of kidney stones and its bad implications on patients there are growing movements for seeking proper management. However, the current treatment modalities like pharmacological and surgical options contribute to many side effects and morbidities [3], that address a dire need to explore new fields like herbal and complementary medicine to ensure safe and effective management with an affordable cost and pay more attention to prevention than treatment.

Many studies have documented the use of antioxidants to protect against nephrotoxicity in both human and animal subjects. These interventions are reported to decrease malondialdehyde (MDA) levels in renal tissue and restore the levels of protective enzymes [9]. Moreover, heightened superoxide dismutase (SOD) activity has been suggested to contribute to the prevention of nephrolithiasis by directly affecting renal epithelial cells [20]. Oral antioxidant therapy prior to lithotripsy has also been proposed to mitigate the severity of long-term renal injury induced by shock waves [21]. These studies have encompassed a range of antioxidants including vitamins A, E, C, B6, as well as antioxidant trace elements like selenium and zinc [[22], [23], [24], [25], [26], [27], [28], [29]]. Additionally, Curhan et al. highlighted that increased fluid intake, particularly in the form of fruit juice, such as orange juice, effectively reduces urinary calcium oxalate saturation and enhances urinary citrate excretion [30].

Allium cepa is renowned for its antioxidant effects, primarily attributed to its constituents, especially quercetin. Existing literature consistently demonstrates that utilizing allium cepa in treatments leads to decreased lipid peroxidation and the levels of NADPH, MDA, and NO [31]. Simultaneously, it enhances antioxidant capacity and levels of SOD, catalase (CAT), and glutathione (GSH) [32,33]. Furthermore, its anti-inflammatory effects have been observed across various inflammatory conditions, spanning cardiovascular, gastrointestinal, neuronal, and respiratory disorders [7,34]. These effects are mediated through modulating inflammatory cells, resulting in reduced total white blood cell counts, neutrophils, and eosinophils, along with inhibited chemotaxis mechanisms of human leukocytes [35]. Allium cepa also exerts inhibitory effects on the COX and LOX pathways, preventing the formation of leukotrienes, thromboxanes, prostaglandin E2 (PGE2), and 12-HHT, all contributing to its pronounced anti-inflammatory effect [36,37]. A recent study by AYANNIYI et al. highlighted the potential anti-inflammatory and mucosal protective effect of Allium cepa peel extract in acetylsalicylic acid-induced gastric inflammation [38].

One notable aspect explored regarding the effect of allium cepa is its immunomodulatory potential, largely attributable to its components, particularly quercetin. This component has been found to reduce Th2 cytokines (IL-4, IL-5, IL-13) as well as IL-6, IL-8, IL-10, IL-1b, TNF-a, and IgE levels [39], while increasing CD4 cells and IFN-c levels, indicating its stimulatory effect on Th1 and inhibitory effect on Th2 activity [40]. Aligning with these existing literature findings, our current study exhibited improved kidney function tests and glomerular filtration function in the group receiving allium cepa extract. This was corroborated by immunohistochemistry results, revealing a significant reduction in TNF and BAX markers in renal tissue. Mast cells, prominent contributors to immunomodulatory and inflammatory effects, remain a topic of debate in terms of their protective or exacerbating role. While they possess the ability to enhance tissue remodeling and counter fibrosis-promoting mechanisms, they can also trigger undesirable responses leading to kidney tissue damage [41]. The present study revealed lesser infiltration of activated mast cells in the allium cepa extract receiving group compared to the EG group.

Notably, prior reports have indicated that the nucleation of calcium oxalate monohydrate crystals within nephron lumens, coupled with crystal-cell interactions, leads to an elevation in the expression of the OPN gene [42]. Consequently, this gene serves as a tool for assessing the efficacy of drugs in combatting stone formation [43]. In our ongoing study, we found that OPN expression was lowest in the treated group, signifying diminished kidney capacity for crystal adhesion and stone formation. This observation aligns with the histopathological results in our study, revealing fewer instances of stone formation and reduced damage to distal convoluted tubules. Although most studies have reported an increase in OPN levels in kidney stone diseases, a distinct debate persists regarding its precise role—whether it promotes macrophage-mediated renal injury or acts protectively through anti-apoptotic and anti-fibrotic effects [44].

Yamaguchi's study highlighted the essential role of regucalcin in regulating intracellular calcium transport, DNA synthesis, activity of cell signaling-related enzymes, and apoptotic cell death in kidney tissue. Moreover, it emphasized that suppressed regucalcin gene expression might contribute to the development of renal failure [45]. With the role of regucalcin in mind, our present study revealed a heightened expression of this gene in the group receiving allium cepa extract compared to the EG group.

## 5 Conclusion

Administration of allium cepa extract serves to prevent excessive kidney stone formation while enhancing kidney function in rats exposed to crystal formation. This effect is mediated through the extract's anti-inflammatory, antioxidant, and immunomodulatory properties. Furthermore, it promotes protective markers in renal tissue, instigating anti-apoptotic and antifibrotic effects. In essence, our study establishes that the administration of allium cepa extract yields a protective impact against renal stone formation and offers significant benefits in ameliorating renal injury associated with kidney stone diseases. Consequently, a pressing need exists for further clinical investigations to validate the protective and therapeutic potential of allium cepa in patients afflicted with kidney stone diseases.

## Funding

This project was funded by the Researchers Supporting Project number (RSPD2023R716), 10.13039/501100002383King Saud University, Riyadh, Saudi Arabia.

## Institutional review board statement

The experimental protocol was approved by Zagazig university institutional animal care and use committee (ZU-IACUC) with number ZU-IACUC/2/F/65/2022.

## Declaration of competing interest

The authors declare that they have no known competing financial interests or personal relationships that could have appeared to influence the work reported in this paper.
